# Integrin-alpha10 drives tumorigenesis in sarcoma

**DOI:** 10.18632/oncoscience.350

**Published:** 2017-04-26

**Authors:** Tomoyo Okada, Samuel Singer

**Affiliations:** Memorial Sloan Kettering Cancer Center, New York, NY 10065, USA

**Keywords:** sarcoma, integrins, TRIO, RAC, mTORC2

Sarcomas represent a heterogeneous group of malignancies of mesenchymal origin with more than 70 histological types. Sarcomas are genetically highly heterogeneous, but they can be classified into two broad groups, one with simple karyotypes (e.g. a chromosomal translocation) and the other with complex genomic alterations and high chromosomal instability [1]. One of the most common complex types is myxofibrosarcoma, which is highly diverse in genetics and clinical outcomes. Thirty to forty percent of the patients die of distant metastasis to lung [2]. Because of the genomic complexity, it has been challenging to identify the molecular drivers and potential therapeutic targets. Unsupervised analysis of gene expression profiles from 64 patients with well-characterized myxofibrosarcomas (primary, untreated high-grade tumors) revealed two subgroups that differ greatly in clinical outcomes. Among the differentially expressed genes, ITGA10 (encoding integrin-α10) was the gene most associated with the risk of metastasis and decreased survival. In addition, ITGA10 expression was significantly higher in metastatic tumors than in primary tumors. These data indicate that integrin-α10 could be a prognostic marker and a critical driver of myxofibrosarcomagenesis [3].

Integrin-α10 belongs to the collagen-binding integrin subfamily, consisting of α1, α2, α10, and α11, all of which form heterodimers with β1 [4]. Unlike the widely expressed α1 and α2, α10 expression is restricted to chondrocytes and a subset of mesenchymal stem cells and fibroblasts. Patient-derived myxofibrosarcoma cell lines strongly depend on integrin-α10 for growth and survival, while normal mesenchymal cells do not [3]. ITGA10-deleted mice are viable and have a normal lifespan with only a mild phenotype in cartilage development, suggesting that integrin-α10 plays a minor role in normal mesenchymal cell growth and survival in adult tissues [5]. Several other collagen-binding integrins have been associated with various types of cancer, but their reported pro-tumorigenic roles are mainly in invasion and migration, which is the primary cellular function of integrins in normal as well as transformed cells [4]. Besides the tumor-autonomic roles, integrin-α11, another mesenchymal-restricted collagen-receptor integrin has well-demonstrated pro-tumorigenic roles in non-tumor cells in the tumor microenvironment, such as cancer-associated fibroblasts. Among all the collagen-associated integrins, integrin-α10 is the least studied for roles in cancer, and its role seems context-dependent, as one report described underexpression associated with tumor promotion, while in melanoma, ITGA10 overexpression was suggested to promote migration of tumor cells but was dispensable for survival [6]. The remarkably strong dependency on integrin-α10 in myxofibrosarcoma cells indicates an unanticipated role as a driver in sarcomagenesis and represents a tumor-specific vulnerability that provides a promising therapeutic target. The narrow tissue specificity of integrin-α10 suggests that targeted agents could have minimal toxicity on normal tissues [3].

Canonical “outside-in” integrin signaling activates FAK and SRC family kinases. Integrin-α10 in myxofibrosarcoma, however, activates RAC/PAK and AKT upon the collagen binding, and not FAK or SRC. Integrin-α10 is essential for AKT and PAK activities in tumor cells but not in normal mesenchymal stem cells. Some pro-oncogenic integrins are known to cooperate with oncogenic receptor tyrosine kinases, including ERBB2 and MET [7]. Even though MET is upregulated in a subset of myxofibrosarcomas, it does not seem to mediate the survival signaling of integrin-α10, because a MET inhibitor, crizotinib, does not cause major growth suppression or apoptosis in myxofibrosarcoma cells [3]. Interestingly, knockdown of integrin-α10 does not impair myxofibrosarcoma cells' adherence to collagen matrix (presumably because the cells express integrin-α1 and integrin-α2). Nevertheless, collagen binding to integrin-α10 is required for PAK and AKT activation. Additionally, integrin-α10-deficient cells die without compromising cell adhesion, indicating that the dependence on integrin-α10 pertains to signaling and not cell adhesion. Furthermore, depletion of either integrin-α1 or integrin-α2 does not inhibit growth, induce apoptosis, or affect AKT and PAK activities. Thus, only collagen binding to integrin-α10 can initiate tumor-specific signaling towards PAK and AKT for the survival of tumor cells [3].

A copy number alteration study of the same 64 myxofibrosarcomas revealed frequent amplification of a chromosome 5p region that carries the oncogenes *TRIO* and *RICTOR* [3], [8]. TRIO is a guanine nucleotide exchange factor that activates RAC. RICTOR an AKT kinase that is an essential subunit of mTORC2. Several lines of evidence demonstrate that integrin-α10 sends its tumor-specific survival signal by activating RAC/PAK via TRIO and by activating AKT via RICTOR [3]. First, knockdown of TRIO/RAC and RICTOR phenocopied ITGA10 deletion in the tumor cells. Second, constitutively active mutants of RAC/PAK and AKT can rescue the integrin-α10 knockdown–induced cell death. Finally, upon ligand engagement, integrin-α10 forms a complex with TRIO and RICTOR but not RAPTOR (an mTORC1 component). An analysis of clinical outcomes revealed a strong association of high TRIO and RICTOR expression with adverse prognosis and risk of metastasis only in patients with ITGA10-high tumors, further supporting the functional link between ITGA10 and TRIO/RICTOR.

Selective inhibitors of RAC (EHop-016), PAK (IPA3), and mTORC (INK128) exerted anti-tumor effects in vitro and in mice, the latter in both xenograft tumors and metastatic growth in the lung. The RAC and mTORC inhibitors had highest efficacy when combined, demonstrating that integrin-α10 downstream signaling is targetable [3]. Together, these findings reveal a remarkable and unanticipated role for targeting the tumor-specific vulnerability created by integrin-α10 signaling in myxofibrosarcoma. They also suggest that RAC/PAK inhibitors alone or in combination with mTOR/AKT inhibitors warrant further investigation for patients with advanced or inoperable myxofibrosarcoma. In fact, these results have already led to the inclusion of MXF/PMFH patients in an ALLIANCE-sponsored phase I/randomized phase II study of MLN0128 (INK128) vs. pazopanib in patients with locally advanced, unresectable, and/or metastatic sarcoma.

## References

[1] Taylor BS (2011). Nat Rev Cancer.

[2] Lee AY (2016). Surg Oncol.

[3] Okada T (2016). Cancer Discov.

[4] Zeltz C (2016). Sci.

[5] Bengtsson T (2005). J Cell Sci.

[6] Wenke AK (2007). Cell Oncol.

[7] Desgrosellier JS (2010). Nat Rev Cancer.

[8] Li CF (2012). Clin Cancer Res.

